# Low-dose whole-spine imaging using slot-scan digital radiography: a phantom study

**DOI:** 10.1186/s12880-023-00971-1

**Published:** 2023-01-30

**Authors:** Shigeji Ichikawa, Hiroe Muto, Masashi Imao, Takashi Nonaka, Kouji Sakekawa, Yasutaka Sato

**Affiliations:** 1 Suzuka University of Medical Science, Graduate School of Health Science Division of Health Science, 1001-1,Kishioka, Suzuka, Mie 510-0293 Japan; 2 Department of Radiology, Faculty of Health Science, Gunma Paz University, 1-7-1 Tonyamachi, Takasaki, Gunma 370-0006 Japan; 3 Department of Radiological Technology, Fussa Hospital, 1-6-1 Kamidaira, Fussa-ku, Tokyo, 197-0012 Japan; 4 Graduate School of Health Science, Suzuka University of Medical Science, 1001-1, Kishioka, Suzuka, Mie 510-0293 Japan

**Keywords:** Slot-scan technology, Whole spine, Radiation dose, Beam-hardening filter, Whole spine radiography

## Abstract

**Background:**

Slot-scan digital radiography (SSDR) is equipped with detachable scatter grids and a variable copper filter. In this study, this function was used to obtain parameters for low-dose imaging for whole-spine imaging.

**Methods:**

With the scatter grid removed and the beam-hardening (BH) filters (0.0, 0.1, 0.2, or 0.3 mm) inserted, the tube voltage (80, 90, 100, 110, or 120 kV) and the exposure time were adjusted to 20 different parameters that produce equivalent image quality. Slot-scan radiographs of an acrylic phantom were acquired with the set parameters, and the optimal parameters (four types) for each filter were determined using the figure of merit. For the four types of parameters obtained in the previous section, SSDR was performed on whole-spine phantoms by varying the tube current, and the parameter with the lowest radiation dose was determined by visual evaluation.

**Results:**

The parameters for each filter according to the FOM results were 90 kV, 400 mA, and 2.8 ms for 0.0 mm thickness; 100 kV, 400 mA, and 2.0 ms for 0.1 mm thickness; 100 kV, 400 mA, and 2.8 ms for 0.2 mm thickness; and 110 kV, 400 mA, and 2.2 ms for 0.3 mm thickness. Visual evaluation of the varying tube currents was performed using these four parameters when the BH filter thicknesses were 0.0, 0.1, 0.2, and 0.3 mm. The entrance surface dose was 59.44 µGy at 90 kV, 125 mA, and 2.8 ms; 57.39 µGy at 100 kV, 250 mA, and 2.0 ms; 46.89 µGy at 100 kV, 250 mA, and 2.8 ms; and 39.48 µGy at 110 kV, 250 mA, and 2.2 ms, indicating that the 0.3-mm BH filter was associated with the minimum dose.

**Conclusion:**

Whole-spine SSDR could reduce the dose by 79% while maintaining the image quality.

## Background

Slot-scan digital radiography (SSDR; Shimadzu, Kyoto, Japan) can remove scatter grids and automatically vary beam-hardening copper filters (BH filters). The filter material is copper, which may reduce X-rays in the low-energy region, thereby reducing the dose.

X-rays are incident perpendicular to the subject or patient such that seamless images with little distortion can be obtained, which is useful for alignment measurement in orthopedics and preoperative and postoperative evaluations. During imaging, the X-ray tube and flat panel detector move simultaneously from the head to the foot at the same speed while delivering X-rays. X-rays are delivered in the form of a beam through a narrow 4-cm slit. The X-ray preparation is completed by confirming the starting position (head side) and the ending position (foot side) with X-ray fluoroscopy. After acquisition, dozens of images acquired using the workstation are superimposed and combined into a single long image. The scatter grid is attached and detached manually, and the BH filter is controlled by a console at hand [1–7].

In general, the image quality is dependent on tube voltage, tube current, and exposure time. The auxiliary factors include scatter grids, filters, distance, and image processing. There is no fixed method to reduce the dose; however, the characteristics of the device and the tools associated with the device can be used to reduce the radiation dose [8–22]. In this study, the parameters of low-dose whole-spine imaging were investigated.

## Methods

Ethical approval was obtained from the Fussa Hospital Ethics Review Committee (Number: 41). The system, phantom, and dosimeter used are shown in Table 1.

**Table 1:** Slot-scan digital radiography equipment and parameters

Imaging unit	SONIALVISION G4; Shimadzu, Kyoto, Japan
High-voltage equipment	DR-300
Flat panel detector (inch)	17
Pixel size (µm)	139
Focus-receptor distance (cm)	110, 120, 150
Grid ratio	10
Beam-hardening filter (copper) (mm)	0.1, 0.2, 0.3
Tube voltage (kV)	40–150 (Fluoroscopy: 50–125)
Tube current (mA)	10-1000 (Fluoroscopy: 0.3–20.0)
Exposure time (s)	0.001–10.0
Density resolution (bits)	16
Whole-body phantom	PBU-60; Kyoto Kagaku, Kyoto, Japan
Dosimeter	Ray Safe X2, Unfors Ray Safe AB, Billdal, Sweden

First, the scatter grids were removed, a BH filter (0.0, 0.1, 0.2, or 0.3 mm) was inserted, and the tube voltage (80, 90, 100, 110, or 120 kV) and exposure time were adjusted to create 20 different parameters that would provide equivalent image quality. The tube current was kept constant at 400 mA.

The Shimadzu Sensitivity (SS) value, which is a sensitivity index used to stabilize the density that was used in Shimadzu’s equipment, was used in this study to obtain equivalent image quality. The SS values are similar to the S values used in X-ray digital imaging, but the concept differs slightly depending on the equipment of the manufacturer.

An SS value of 200 is defined as the value obtained when 2.58 × 10^−7^ C/kg of radiation reaches the detector at a tube voltage of 80 kV. The optimal image has an SS value of 200 to 300. In this study, the SS value was adjusted to be in the range of 250 ± 20%. The 20 different parameters are listed in Table 2.

**Table 2:** Measurements obtained from the imaging parameters

BH filter (mm)	Tube voltage (kV)	Tube current (mA)	Exposure time (ms)	ESD (µGy)	CNR	FOM
0.0	80	400	4.5	151.01	0.012476	10.3073 × 10^−7^
	90	400	2.8	128.51	0.011805	10.8441 × 10^−7^
	100	400	2.0	110.87	0.010008	9.0340 × 10^−7^
	110	400	1.4	104.02	0.009008	7.8011 × 10^−7^
	120	400	1.0	93.11	0.008111	7.0657 × 10^−7^
0.1	80	400	6.3	118.60	0.009911	8.2829 × 10^−7^
	90	400	3.2	88.99	0.008880	8.8610 × 10^−7^
	100	400	2.0	69.76	0.008497	10.3496 × 10^−7^
	110	400	1.6	67.13	0.006588	6.4653 × 10^−7^
	120	400	1.2	62.50	0.005658	5.1221 × 10^−7^
0.2	80	400	7.1	101.04	0.008222	6.6906 × 10^−7^
	90	400	4.5	82.42	0.007534	6.8868 × 10^−7^
	100	400	2.8	66.39	0.006834	7.0347 × 10^−7^
	110	400	1.8	60.82	0.005984	5.8876 × 10^−7^
	120	400	1.2	55.50	0.005572	5.5941 × 10^−7^
0.3	80	400	9.0	85.55	0.006754	5.3322 × 10^−7^
	90	400	5.6	72.08	0.006356	5.6047 × 10^−7^
	100	400	3.2	66.01	0.006141	5.7131 × 10^−7^
	110	400	2.2	60.04	0.006026	6.0481 × 10^−7^
	120	400	1.4	55.99	0.005711	5.8252 × 10^−7^

The tube current is constant at 400 mA on parameters

*BH* beam hardening, *ESD* entrance surface dose, *CNR* contrast-to-noise ratio, *FOM* figure of merit

### Method 1

**Fig. 1:**
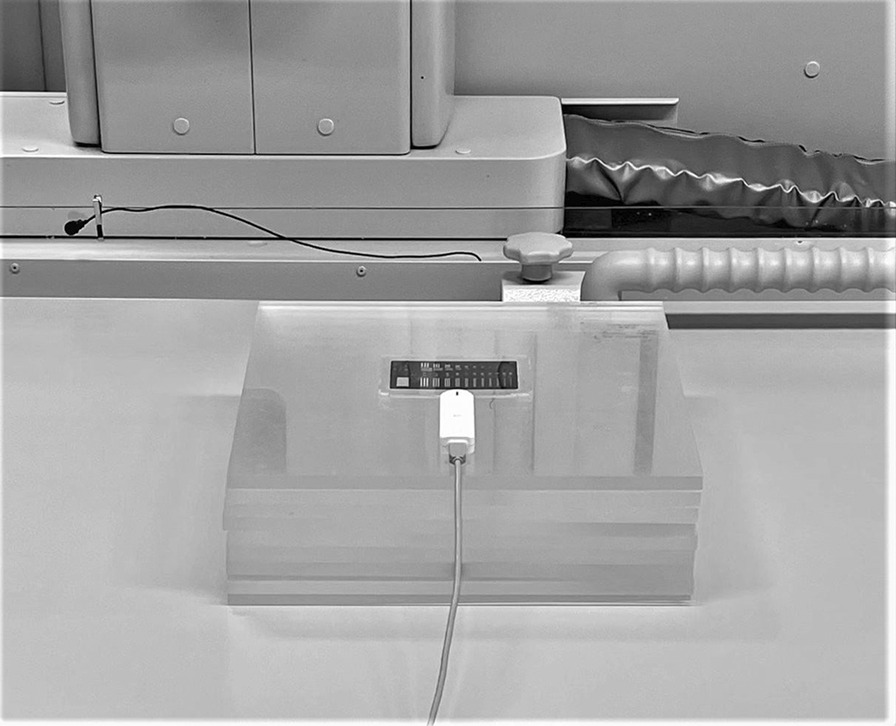
The layout of slot-scan radiography with 20 different parameters is shown. The X-ray chart and dosimeter probe were placed on an acrylic phantom (20 cm thick), and slot-scan radiography was performed to obtain the chart image and dose

**Fig. 2:**
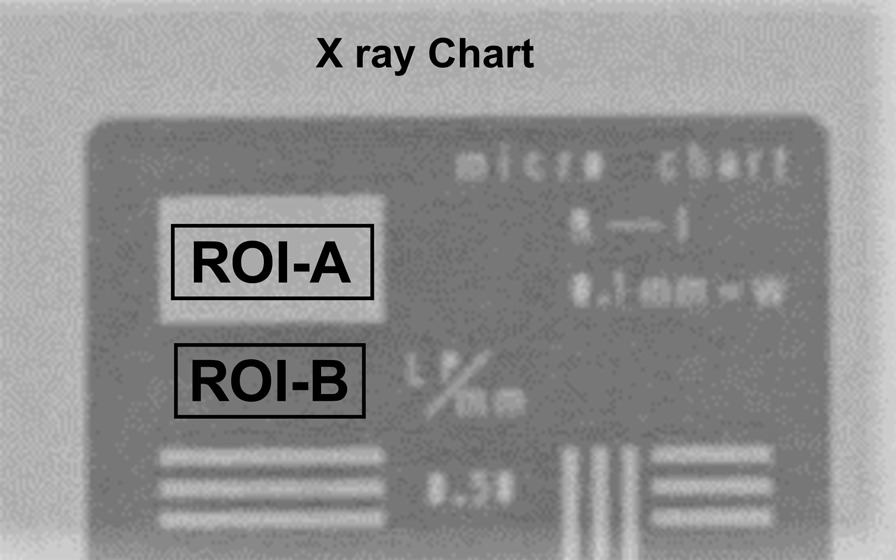
Region of interest (ROI) settings for contrast-to-noise ratio (CNR) measurements. Two ROIs were established on the X-ray chart

SSDR was performed using 20 different parameters on an acrylic phantom (20 cm thick) with an X-ray chart and dosimeter probe placed on it, and images and entrance surface dose (ESD) were measured (Fig. 1; Table 1). From the images acquired, the “Mean ROI-A” and “Mean ROI-B” of the region of interest were defined in ImageJ (ver. 1.41; National Institutes of Health, Bethesda, MD), as shown in Fig. 2, and the standard deviation (SD) for noise (N) was used to obtain the contrast-to-noise ratio (CNR) (Eq. (1)).

1 $${\text{CNR}}=\frac{{\text{Mean ROI-A}}-{\text{Mean ROI-B}}}{{\text{SD}}}$$

The figure of merit (FOM) was then determined for each filter using Eq. (2) to determine the best scoring parameters (four types).

2 $${\text{FOM}}=\frac{({\text{CNR}})^{2}}{{\text{ESD}}}$$

FOM is a widely used tool to evaluate or compare the performance of a device, material, or procedure. As a tool for the quantitative evaluation of digital images, it is used for the comparative evaluation of X-ray equipment and parameters in the field of radiography. It is defined as the ratio of the X-ray dose to the square of the CNR, which indicates the image quality; a higher score indicates a better evaluation [23–25]. FOM was used as the slot-scan images are acquired through a narrow 4-cm slit, leading to less scattered radiation and acquisition of images that maintain contrast even at high voltage. Using this feature, it would be possible to find low-dose parameters by setting a high tube voltage.

### Method 2

For each of the four parameters of each filter obtained in Method 1, the tube current was reduced to 400, 320, 250, and 125 mA, and the whole-spine phantoms were imaged for ESD and visual evaluation (Table 1).

**Fig. 3:**
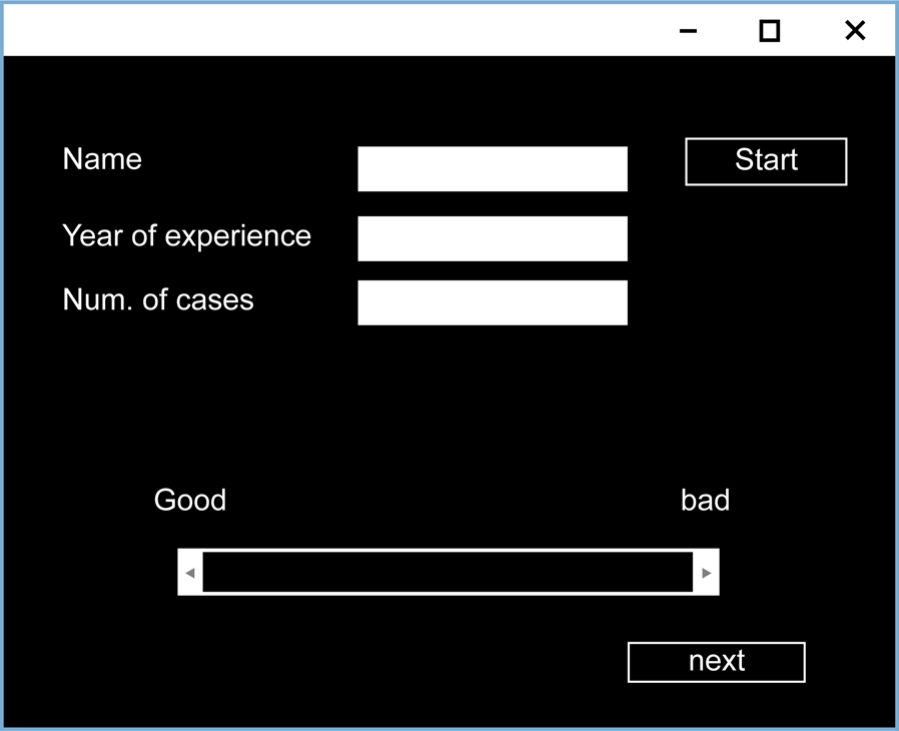
Visual analog scale (VAS) used for visual assessment. Scores were given by moving the scale from good to bad (continuous confidence method). Five physicians (four orthopedic surgeons and one radiologist) oversaw the evaluation. The purpose of the test was fully explained prior to the evaluation, which was conducted in the same environment, under the same conditions (time, room light, monitors)

A visual evaluation was performed using a visual analog scale (VAS) for each BH filter (Fig. 3). The VAS is a tool for objectively evaluating subjective ratings that are difficult to measure. It is one of the most frequently used methods in the assessment of pain [26–29]. When responding to items on the VAS, respondents select a position on a continuous line connecting two endpoints (good–bad). The specific evaluation method was based on whether the phantom was diagnostically observable from the cervical spine to the lumbar spine on imaging. The VAS scale was used to score the phantoms as “good” if observable and “bad” if unobservable. The evaluators were five physicians (four orthopedic surgeons and one radiologist) who understood and agreed with the purpose of the study. Analyses were performed by multivariate analysis (non-parametric, three-group comparison, Kruskal–Wallis test) and multiple comparisons (Steele–Drewas method) using Easy E (EZR, ver. 1.55) [30].

## Results

**Fig. 4:**
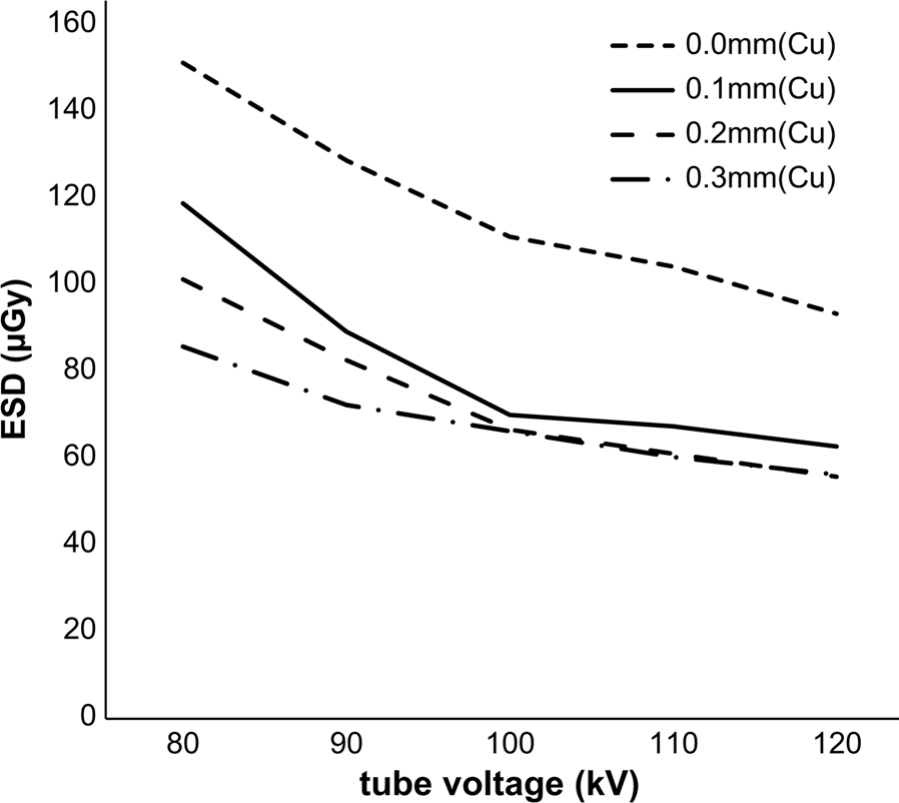
Entrance surface dose (ESD) for each filter in phantom imaging with 20 different parameters

**Fig. 5:**
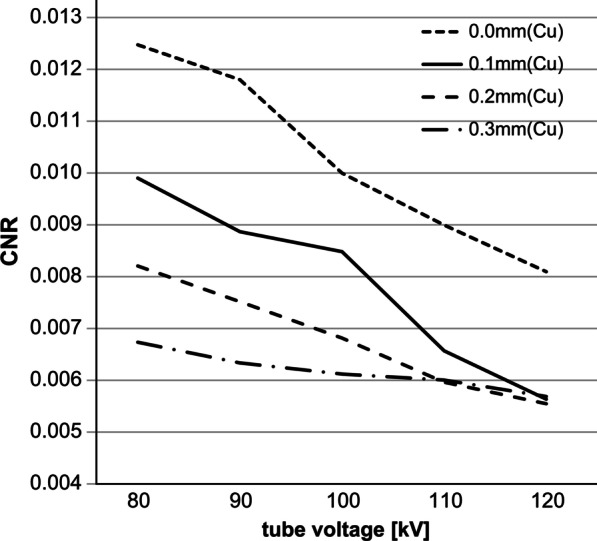
Contrast-to-noise ratios (CNRs) based on 20 different parameters (X-ray chart analysis)

**Fig. 6:**
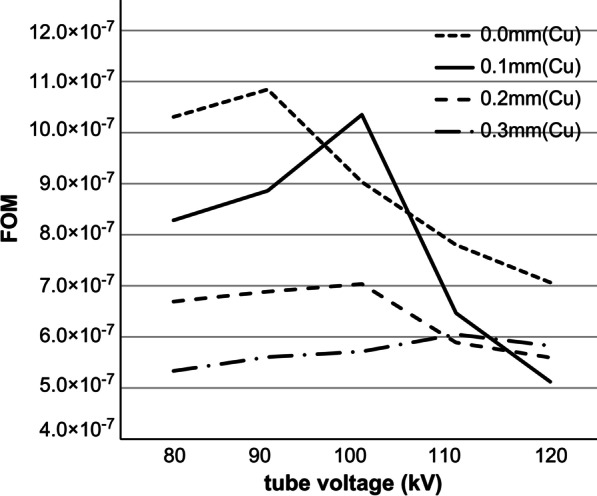
ESD, CNR, and FOM obtained from 20 different parameters. ESD: Entrance surface dose; CNR: contrast-to-noise ratio; FOM: figures of merit

In Method 1, the ESD was the highest at 151.01 µGy when the filter thickness was 0.0 mm and the tube voltage was 80 kV; the ESD was the lowest at 55.50 µGy when the filter thickness was 0.2 mm and the tube voltage was 120 kV, showing a decrease of about 60%. The ESD tended to decrease as the tube voltage increased (Table 2; Fig. 4). The CNR was best at 0.012476 when the filter thickness was 0.0 mm and the tube voltage was 80 kV, and it was the lowest at 0.005572 when the filter was 0.2 mm and the tube voltage was 120 kV. The CNR tended to decrease as the tube voltage between the filters increased (Table 2; Fig. 5). When the BH filter thickness was 0.0 mm, the FOM was the highest at 10.841 × 10^−7^ at a tube voltage of 90 kV. For a BH filter thickness of 0.1 mm, the FOM was the highest at 10.3496 × 10^−7^ at a tube voltage of 100 kV. For a BH filter thickness of 0.2 mm, the FOM was the highest at 7.0347 × 10^−7^ at a tube voltage of 100 kV. For a BH filter thickness of 0.3 mm, the FOM was the highest at 6.0481 × 10^−7^ at a tube voltage of 110 kV (Fig. 6). Method 2, which used the abovementioned four parameters, was considered for use since the highest score in the FOM results can be defined as the optimal parameter.

**Fig. 7:**
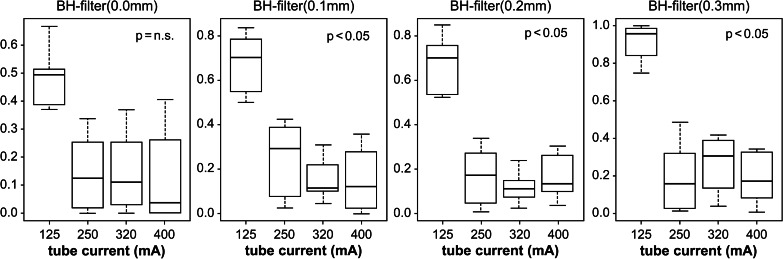
Visual evaluation results for each filter. When the filter thickness is 0.0 mm, no significant difference was observed between the different tube currents. For filter thicknesses of 0.1, 0.2, and 0.3 mm, significant differences were observed for tube currents of 125, 250, 320, and 400 mA (multiple comparison, Steel–Dwass method)

**Fig. 8:**
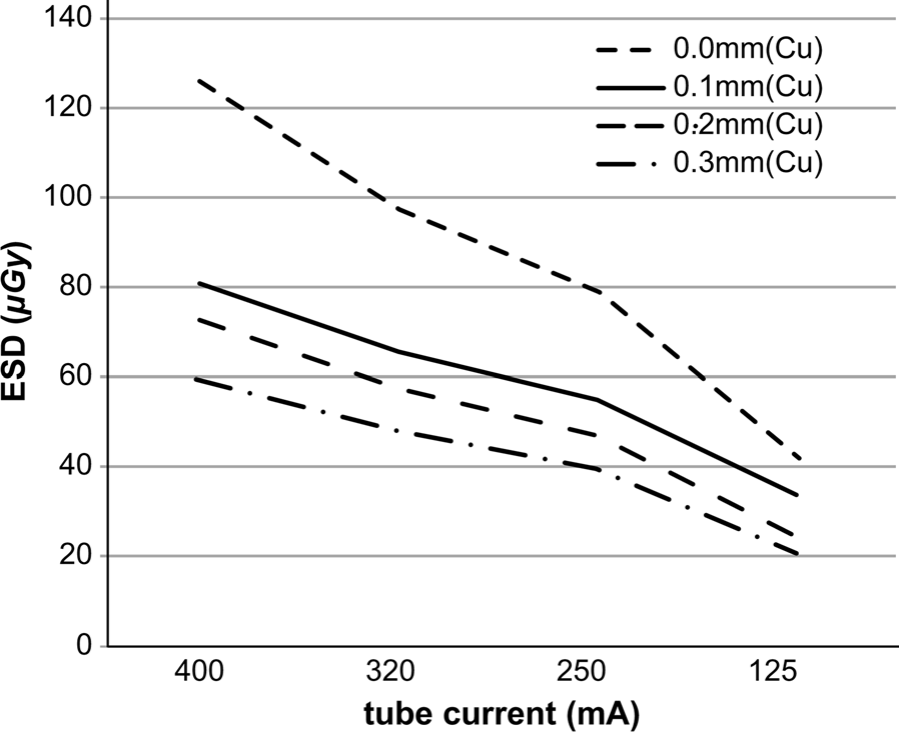
Entrance Surface Dose (ESD) for the 16 parameters shown in Table 3

As a result of Method 2, Table 3 shows the 16 parameters obtained by reducing the tube current for the four parameters obtained from Method 1. Using these parameters, a whole-body phantom was photographed, and the results of the visual evaluation and dosimetry are shown in Figs. 7 and 8. In the visual evaluation, no significant difference was observed in the images with respect to the change in tube current when the filter was 0 mm (*p* = 0.06). When the filter was 0.1, 0.2, and 0.3 mm, significant differences were observed in the 125-mA current range and other ranges (*p* < 0.05) (Fig. 7). The ESD decreased with decreasing tube current (Fig. 8).

**Table 3:** Parameters obtained from varying tube current for each filter (16 types)

BH filter (mm)	Tube voltage (kV)	Tube current (mA)	Exposure time (ms)
0.0	90	400/320/250/125	2.8
0.1	100	400/320/250/125	2.0
0.2	100	400/320/250/125	2.8
0.3	110	400/320/250/125	2.2

*BH* beam hardening

## Discussion

The parameters obtained by FOM aim to reduce the dose by varying the tube voltage. This system is characterized by low scattered rays and good contrast images even in the high-voltage region as imaging is performed with a 4-cm wide slit; this could be the reason why the FOM results were better in the high-voltage region.

The copper filter also attenuates X-rays in the low-energy region. Typically, aluminum or copper is used to attenuate X-rays in the low-energy region. Copper has a larger atomic number and is more effective in attenuating X-rays than aluminum, thereby lowering the overall dose without changing the effective dose. Since this study was conducted in the high-voltage region, the effect of copper can be considered to be demonstrated. Furthermore, it was considered that parameters with a good balance between the image quality and dose were obtained using FOM, compared with the output obtained when the parameters recommended by the manufacturer were used in the past.

As for the radiation dose of the whole-body phantom during visual evaluation, the dose decreased at a constant rate when the tube current was decreased to 400, 320, 250, and 125 mA. This is because the tube current is proportional to the radiation dose.

Visual evaluation using the VAS showed no significant difference when the tube current was lowered if the BH filter was 0.0 mm (*p* = 0.06) as the low-energy component is not removed when the BH filter is not used; therefore, it was considered that a decrease in radiation dose does not make a difference in the evaluation.

**Fig. 9:**
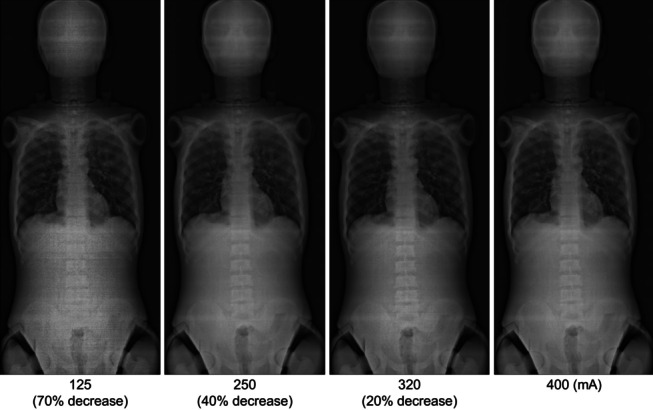
Whole-spine images at a beam-hardening (BH) filter thickness of 0.3 mm. The tube currents were 400, 320, 250, and 125 mA. The tube voltage was 110 kV, and the exposure time was 1.4 ms. The lumbar spine and pelvic region are obscured at a tube current of 125 mA and can be observed at tube currents of 250, 320, and 400 mA

However, a significant difference (*p* < 0.05) was observed at a tube current of 125 mA for 0.1-, 0.2-, and 0.3-mm BH filters. This was considered to be a significant difference at a certain boundary as the low-energy component is removed and the radiation dose is reduced. As a result, the lowest dose was obtained with a 0.3-mm BH filter (Fig. 9).

The lowest radiation (scatter grid uninstalled, 0.3-mm BH filter installed, 110 kV/250 mA/2.2 ms, and ESD of 39.48 µGy) dose was 79% lower than that with the parameters used in conventional examination (scatter grid installed, no filter installed, 85 kV/400 mA/6.3 ms, and ESD of 191.1 µGy; measured beforehand). In addition, there was no difference among the evaluations performed by the physicians involved in the evaluation; therefore, the validity of the evaluation result was considered guaranteed. There is no fixed method for dose reduction.

Although there are many systems for imaging the entire spine, some systems make it difficult to reduce the dose as the scatter grid cannot be removed or a BH filter cannot be installed. In addition, it is assumed that some devices cannot guarantee image quality due to increased scattered radiation at high voltage settings; therefore, SSDR may be useful.

In addition, when considering dose reduction, it is difficult to examine the tube voltage and tube current simultaneously. Therefore, the tube voltage was examined first, and the tube current was examined based on the results of that examination. Under these circumstances, FOM is a useful tool.

Since the main purpose of whole-spine imaging is to measure the alignment and observe the entire spine, some degradation of image quality is considered acceptable. In addition, imaging is performed in all age groups and includes radiosensitive areas, such as the breast, spine, and gonads. Radiation doses should be reduced as much as possible based on the ALARA (“as low as reasonably achievable”) concept. This study is effective as a dose-reduction method. However, this study does not include data based on patient-specific body shape or age, or disease-specific bone density. Therefore, its use in clinical practice requires further study. Notably, the potential for dose reduction is great, and a reduction in exposure dose is expected to significantly reduce the risk from radiation exposure.

## Conclusion

Compared with conventional parameters, a 79% dose reduction was achieved when a 0.3-mm BH filter was used in this study. The image quality was determined to be the same via visual evaluation. During the imaging of the entire spine with SSDR, dose reduction can be achieved without compromising the image quality by using high voltage, low current, removal of scattering grids, and BH filters.

## Data Availability

The datasets used and/or analysed during the current study are available from the corresponding author on reasonable request.

## Abbreviations

BH: Beam-hardening
CNR: Contrast-to-noise ratio
ESD: Entrance surface dose
FOM: Figure of merit
SSDR: Slot-scan digital radiography
VAS: Visual analog scale
